# *BCR::ABL1*-like acute lymphoblastic leukaemia: a single institution experience on identification of potentially therapeutic targetable cases

**DOI:** 10.1186/s13039-023-00645-1

**Published:** 2023-07-03

**Authors:** Anna Płotka, Anna Przybyłowicz-Chalecka, Maria Korolczuk, Zuzanna Kanduła, Błażej Ratajczak, Jolanta Kiernicka-Parulska, Anna Mierzwa, Katarzyna Godziewska, Małgorzata Jarmuż-Szymczak, Lidia Gil, Krzysztof Lewandowski

**Affiliations:** 1 Department of Haematology and Bone Marrow Transplantation, Poznań University of Medical Sciences, Poznań, Poland; 2 Institute of Human Genetics, Polish Academy of Sciences, Poznań, Poland

**Keywords:** *BCR::ABL1*-like acute lymphoblastic leukemia, Cytogenetic analysis, Molecular characteristic, Molecular-targeted therapy

## Abstract

**Background:**

*BCR::ABL1*-like acute lymphoblastic leukaemia (*BCR::ABL1*-like ALL) is characterized by inferior outcomes. Current efforts concentrate on the identification of molecular targets to improve the therapy results. The accessibility to next generation sequencing, a recommended diagnostic method, is limited. We present our experience in the *BCR::ABL1*-like ALL diagnostics, using a simplified algorithm.

**Results:**

Out of 102 B-ALL adult patients admitted to our Department in the years 2008–2022, 71 patients with available genetic material were included. The diagnostic algorithm comprised flow cytometry, fluorescent in-situ hybridization, karyotype analysis and molecular testing with high resolution melt analysis and Sanger Sequencing. We recognized recurring cytogenetic abnormalities in 32 patients. The remaining 39 patients were screened for *BCR::ABL1*-like features. Among them, we identified 6 patients with *BCR::ABL1*-like features (15.4%). Notably, we documented *CRLF2*-rearranged (*CRLF2*-r) *BCR::ABL1*-like ALL occurrence in a patient with long-term remission of previously *CRLF2-*r negative ALL.

**Conclusions:**

An algorithm implementing widely available techniques enables the identification of *BCR::ABL1*-like ALL cases in settings with limited resources.

## Introduction

B-cell acute lymphoblastic leukaemia (B-ALL) is a malignancy resulting from the transformation of a B-cell lineage progenitor cell [1]. The hallmark of B-ALL cases is the presence of genetic abnormalities, including chromosomal rearrangements, DNA copy number variations (CNV) and sequence mutations [2]. The 5th edition of the WHO classification divides B-ALL entity on the basis of refined diagnostic criteria and emphasis on therapeutically and/or prognostically actionable biomarkers [3]. The recently updated classification delineates a newly identified molecular subtype—B-ALL with *BCR::ABL1*-like features as a separate entity. It is characterized by a similar gene expression profile to the ALL with *BCR::ABL1*-fusion, but lacks the *BCR::ABL1* fusion gene [4, 5]. It is exclusive of well-known drivers of B-ALL, including *BCR::ABL1* fusion, *KMT2A* rearrangement, *ETV6::RUNX1* and *TCF3::PBX1* fusions [5]. The prevalence of the *BCR::ABL1*-like ALL is impacted by the age and ethnicity of distinct cohorts and the identification methodology. The incidence increases with age, with a peak in young adults population [6–8]. It is characterized by inferior outcomes due to a high rate of nonresponse to induction therapy, higher relapse risk, lower overall survival rates and the persistence of minimal residual disease (MRD) [9–11].

Diverse genetic alterations dysregulating kinase and receptor signaling are the hallmark of the *BCR::ABL1*-like ALL and can be divided into several classes: (1) alterations activating JAK-STAT pathway signaling (including rearrangements of cytokine receptor-like factor 2 (*CRLF2*) gene, Janus kinase 2 (*JAK2*) gene and erythropoietin receptor (*EPOR*) gene); (2) rearrangements of ABL-class genes (*ABL1*, *ABL2*, *PDGFRα*, *PDGFRβ*, *CSF1R*); (3) Ras pathway mutations (*KRAS*, *NRAS*, *NF1*, *PTPN11*) and other uncommon rearrangements [6, 7, 12].

The underlying molecular changes in the *BCR::ABL1*-like ALL remain of significant interest due to the possibility of incorporation of targeted therapy with tyrosine kinase inhibitors (TKI) and JAK inhibitors [13, 14]. Several ongoing clinical studies are evaluating the effectiveness of addition of targeted therapy to chemotherapy to improve the prognosis [15]. Current scientific efforts concentrate on the identification of molecular targets, and numerous algorithms have been proposed for the recognition of the *BCR::ABL1*-like ALL subtype, including targeted fusion testing, tiered algorithms and broad-based testing [16–19]. Nevertheless, the principal aim of the diagnostic approach is to recognize the underlying genetic feature, since they are determinative for the prognosis and targeted therapy. In smaller, real-world groups with constrained resources, the access to comprehensive sequencing strategy is limited. Hence, in those centers, the testing methods should be tailored.

Herein, we present our experience in the *BCR::ABL1*-like ALL diagnostics. We applied an integrated algorithm which allowed a cost-effective detection of this entity. The frequency and clinical outcome of *BCR::ABL1*-like ALL cases were analyzed and compared with the existing literature data, with a particular emphasis on the potential therapeutic options.

## Materials and methods

### Patients

The study was conducted at the Department of Hematology and Bone Marrow Transplantation of Poznan University of Medical Sciences. Adult patients diagnosed with B-cell ALL treated at our Department in the years 2008–2022 were included (n = 102). Thirty-one patients were excluded from further analysis due to the lack of cytogenetic material or essential clinical data. We performed a retrospective analysis of the clinical data, cytogenetic and molecular characteristics in patients treated in the years 2008–2020 (n = 63). Independently, a prospective analysis of cases diagnosed in the years 2020–2022 was performed (n = 8). This study was conducted in accordance with the Declaration of Helsinki. The study was approved by the Poznań University of Medical Sciences Bioethical Committee (Resolution No. 705/20). 63 patients (88.7%) enrolled in the study were treated with B-ALL protocols according to the Polish Adult Leukemia Group (PALG) guidelines. Remaining patients were treated according to hyper-CVAD protocol.

### Methods

The expression of TSLPR (predictive of the rearrangement of the *CRLF2*) with an anti-TSLP antibody (Invitrogen™, clone 1F11/TSLPR PE) was performed using the 10-color multiparameter flow cytometry method (FCM; BD FacsCanto II Ilyric™) using the strategy of internal negative control.

The karyotype analysis was performed using G banding (GTG). The results were described according to the International System for Human Cytogenetic Nomenclature (ISCN). FISH studies were performed on the interface nuclei using break-apart probes for TCF3::PBX1, CRFL2, JAK2, EPOR, ABL1, ABL2 (Cytocell, Cambridge, UK) and for BCR::ABL1, KMT2A, and PDGFRb (Vysis, IL, USA) and, additionally, for IGH and P2RY8 in the *CRLF2* rearranged (*CRLF2*-r) cases (Cytocell, Cambridge, UK). At least 200 interphase nuclei were scored for each probe by two independent examiners. The cut-off threshold for the *BCR::ABL1*-like FISH probes of > 10% of cells was established.

The analysis of the *JAK2* exon 16 sequence was conducted using DNA extracted from whole-blood leukocytes at the time of diagnosis QIAmp DNA Mini Kit (Qiagen) and high resolution melt analysis (HRMA). For the variant type identification screened by HRMA, Sanger sequencing was applied using the BigDye Terminator v3.1 Cycle Sequencing kit (Applied Biosystems, Thermo Fisher Scientific) and the following primers—forward: 5ʹ-TGCTCCAGTACTTGTGGACTGA-3ʹ and reverse: 5ʹ-CCACTGCCCAAGTAAAGCTTAG-3ʹ.

### Diagnostic algorithm

**Fig. 1:**
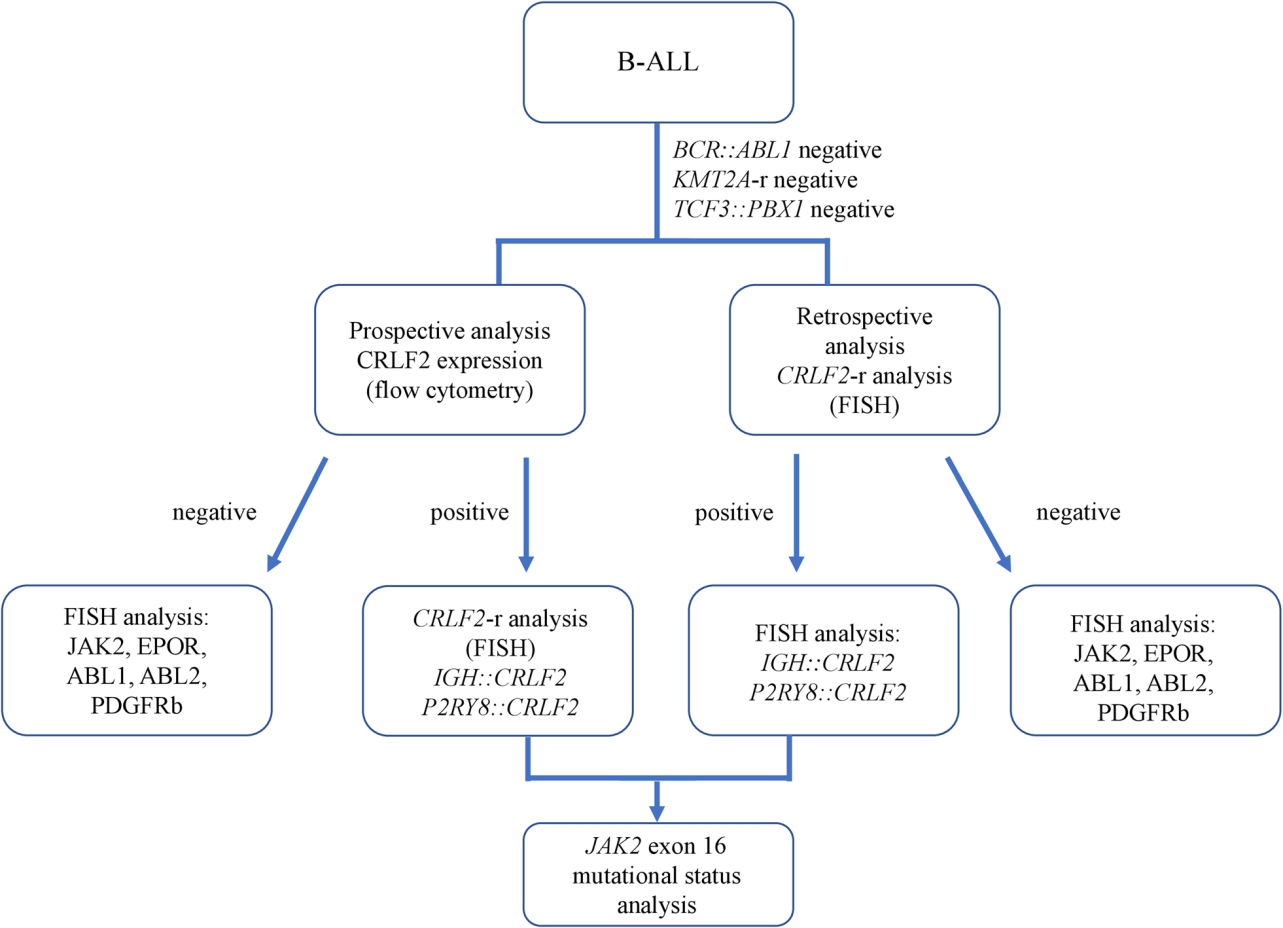
A stepwise algorithm integrating multicolor flow cytometry and fluorescent in situ hybridization implemented in the study

For the identification of *BCR::ABL1*-like ALL cases, we implemented a stepwise algorithm integrating all the above-mentioned techniques (Fig. 1).

Firstly, the presence of the recurring cytogenetic features was evaluated. Patients with recurring cytogenetic lesions were excluded from screening for *BCR::ABL1*-like ALL. The expression of TSLPR was evaluated by FCM (in a prospective analysis only). Patients expressing TSLPR on leukemic blasts were enrolled for the FISH analysis with a CRLF2 break-apart probe. Patients lacking the TSLPR expression were recognized as non-*CRLF2*-rearranged (non-*CRLF2*-r) and subsequently proceeded to the analysis with remaining FISH probes (JAK2, EPOR, ABL1, ABL2, PDGFRb). In a case of retrospective analysis, the patients were primarily examined for the presence of *CRLF2* rearrangement with a FISH probe. In *CRLF2*-r cases, the next step included an analysis with IGH and P2RY8 FISH probes to identify the fusion gene. Non-*CRLF2*-r cases proceeded to the analysis with the remaining FISH probes (JAK2, EPOR, ABL1, ABL2, PDGFRb).

Additionally, all patients with *CRLF2*-r B-ALL with available DNA were enrolled in the analysis of the *JAK2* exon 16 mutational status.

## Results

The median age of the patients at the time of initial diagnosis was 40 (range 18–69 years). Most of the individuals were diagnosed with the B-common phenotype (n = 50). 32 patients from the study group were recognized as B-ALL with recurring cytogenetic abnormalities. The remaining patients (n = 39) were screened for *BCR::ABL1*-like features. Out of the prospectively analyzed subjects (n = 8), we revealed high expression of CRLF2 in 3 cases in FCM. Second step analysis with FISH revealed *CRLF2::IGH* fusion in all 3 patients. In a retrospectively analyzed group, we revealed *CRLF2*-r in one patient and ABL-class genes rearrangements in 2 patients.

**Fig. 2:**
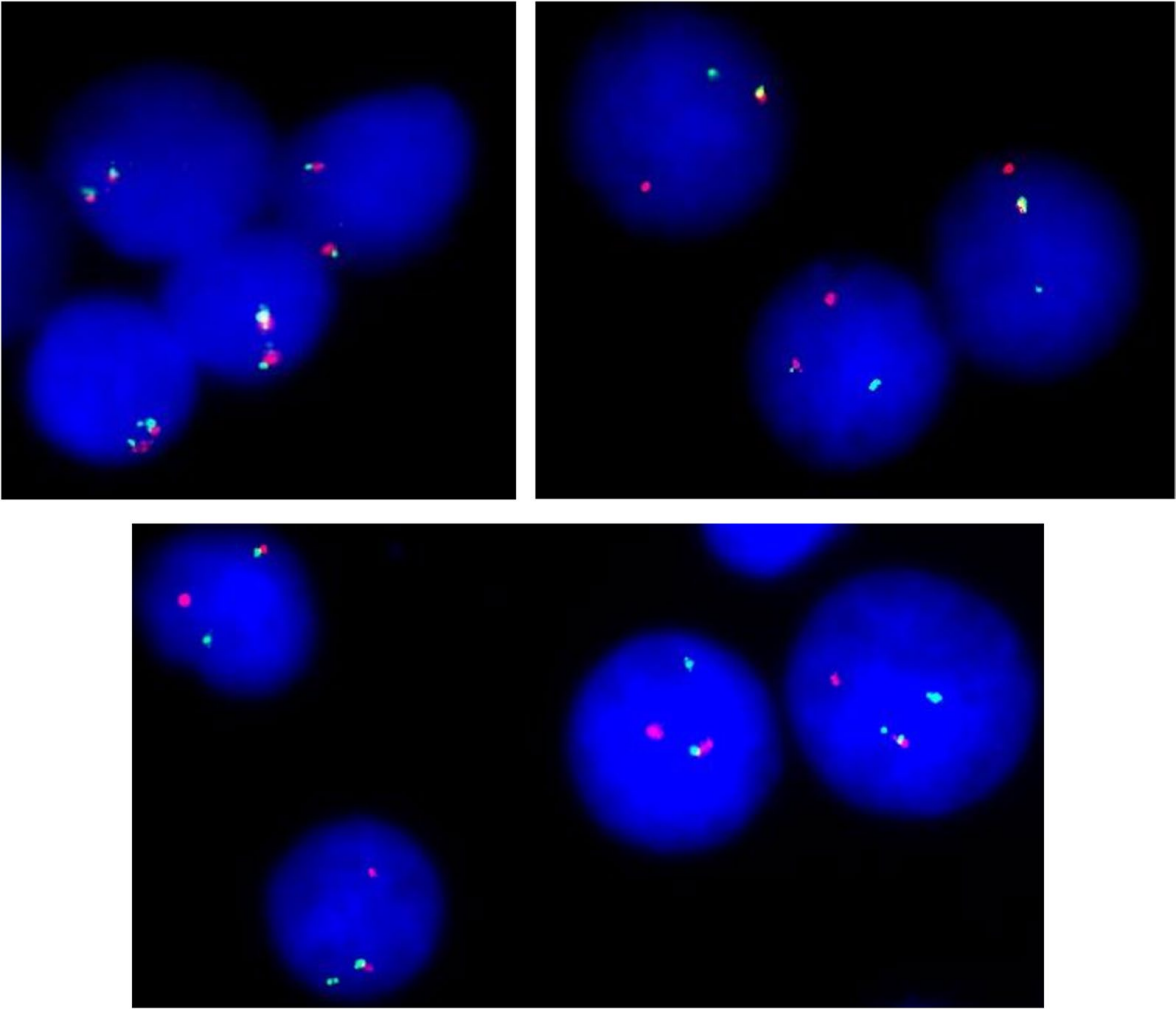
The results of a diagnostic work-up of a patient with *CRLF2*-rearranged *BCR::ABL1-like* ALL which occurred during a relapse after a prolonged remission despite the absence of *CRLF2* rearrangement at the initial diagnosis. Top left side: FISH analysis with CRLF2 break-apart probe (CytoCell®) on leukemic blasts at the initial diagnosis. In the normal cell, 2 fused red/green signals (2 R/G) or 2 yellow signals (2Y) are observed. Top right side: FISH analysis with CRLF2 break-apart probe (CytoCell®) on leukemic blasts at the relapse after prolonged remission (13 years). A translocation resulting in 1R, 1G, 1R/G. Bottom: Second step analysis with IGH probe (CytoCell®). In a normal cell, 2 fused red/green signals (2 R/G) or 2 yellow signals (2Y) are expected. Cells with 1R, 1G, 1R/G are indicative of IGH rearrangement. ALL, acute lymphoblastic leukemia; CRLF2, cytokine receptor-like factor 2; FISH, fluorescent in-situ hybridization

Interestingly, one of the patients was enrolled in the study due to a relapse of B-ALL within 13 years after the treatment with chemotherapy and an allogeneic hematopoietic stem cell transplantation (alloHSCT) in the first complete remission (CR1). Notably, we performed a retrospective analysis on the basis of cytogenetic material obtained at the time of the initial diagnosis, however, the rearrangement of *CRLF2* was absent. The results of the cytogenetic analysis in the relapsed case is presented in Fig. 2.

**Fig. 3:**
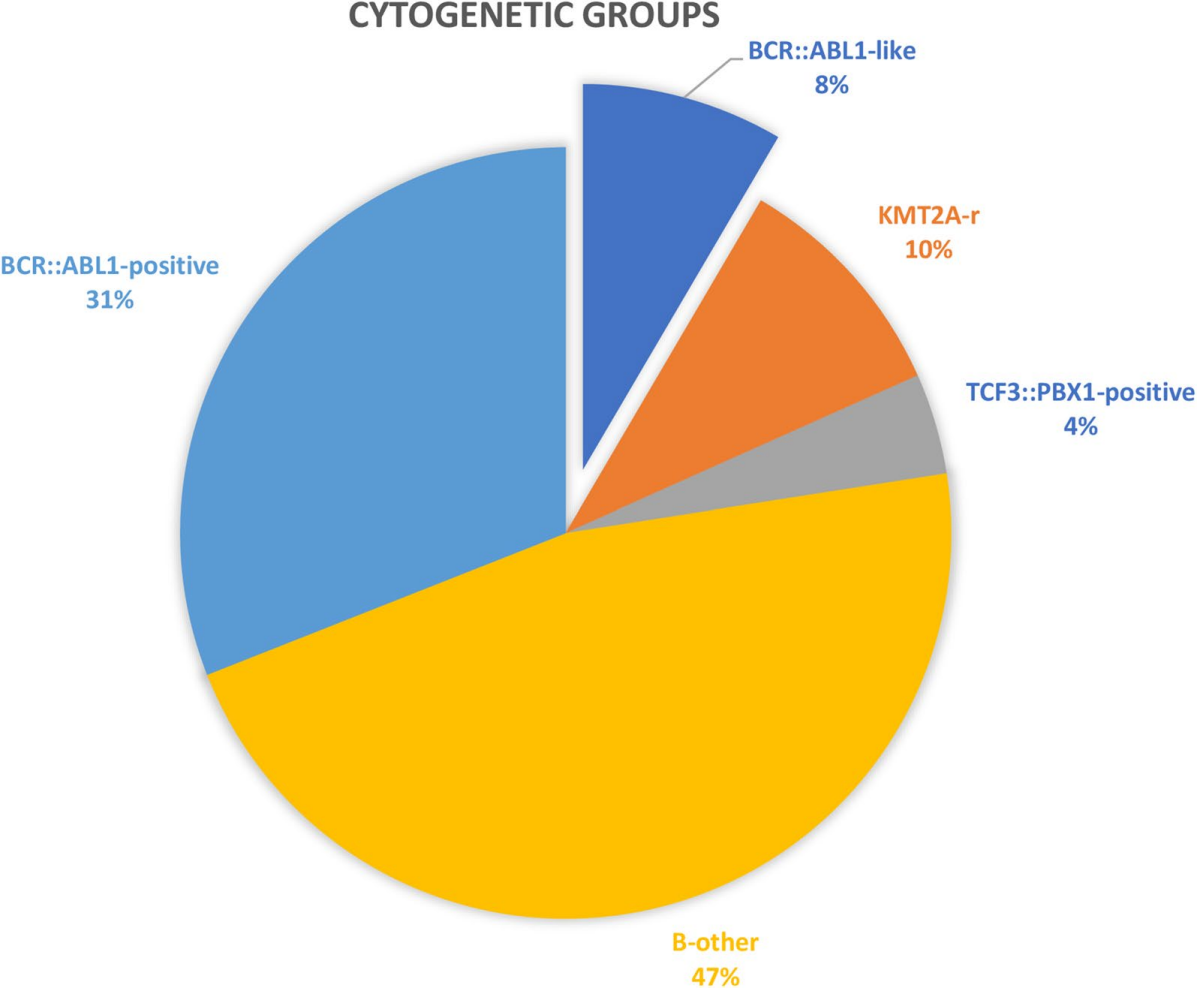
Cytogenetic and molecular characterization of the studied B-ALL patients. The proportion of patients for a particular subgroup of the whole cohort (n = 71)

The incidence of the *BCR::ABL1*-like ALL among patients lacking recurrent cytogenetic features was 15.4% and in the whole study group of B-ALL patients it was 8.5%. Most of the cases were *CRLF2*-r (n = 4; 66.7%). Overall, we distinguished 5 subtypes of B-ALL in the study group: *BCR::ABL1* positive ALL, *BCR::ABL1*-like ALL, ALL with *KMT2A*-r, *TCF3::PBX1* positive ALL and other B-ALL. In Table 1 we present a brief summary of clinical characteristics of distinguished cytogenetic subtypes of B-ALL. In Table 2 we present the clinical characteristics of *BCR::ABL1*-like ALL patients. The incidence of distinct entities is presented in Fig. 3.

**Table 1:** Clinical and laboratory characteristics of distinct cytogenetic subgroups of the studied patients with B-ALL (n = 71)

Parameter	*BCR::ABL1*-like n = 6	Ph-negative other n = 33	Ph-negative *KMT2A*-r n = 7	Ph-negative *TCF3::PBX1*-positive n = 3	*BCR::ABL1*-positive n = 22
Male; n (%)	5 (83.3%)	25 (75.6%)	0	1 (33.3%)	8 (36.4%)
Age (years)					
Median (range)	31.5 (21–55)	32 (18–69)	35 (29–59)	49 (24–55)	43 (19–68)
Immunophenotype					
B-common	5	23	0	1	21
Pro-B	1	8	6	0	1
Pre-B	0	2	0	1	0
NOS	0	0	1	1	0
Aberrant expression of myeloid antigens					
n (%)	4 (66.7%)	10 (30.3%)	0	0	13 (59.1%)
WBC (× 10^9^/L)					
Median (range)	38.2 (9.4–220)	4.7 (0.5–208)	44.5 (4.8–259.5)	3.35 (2.6–34)	11.5 (0.9–131.1)
CNSi; n (%)	1 (9.1%)	4 (12.1%)	4 (57.1%)	1 (33.3%)	6 (27.3%)
Response to induction					
CR MRD^–^; n (%)	4 (66.7%)	18 (54.5%)	7 (100%)	1 (33.3%)	12 (54.5%)
CR MRD^+^; n (%)	2 (33.3%)	4 (12.1%)	0	2 (66.7%)	8 (36.4%)
AlloHSCT; n (%)	3 (50%)	16 (48.5%)	4 (57.1%)	2 (66.7%)	14 (63.65%)
Alive; n (%)	2 (33.3%)	15 (45.5%)	2 (29%)	1 (33.3%)	15 (68.2%)

*B-ALL* B cells acute lymphoblastic leukemia, *NOS* not otherwise specified, *WBC* white blood cells, *CNSi* central nervous system involvement, *Myeloid antigens* CD13, CD33, CD36, CD117, *CR* complete remission, *MRD* minimal residual disease, *alloHSCT* allogeneic stem cells transplantation; complex karyotype: ≥ 3 unrelated (acquired) chromosomal abnormalities; another result: abnormal but non-complex karyotype

**Table 2:** Characteristics and clinical outcome of patients with *BCR::ABL1*-like ALL

Age at diagnosis/gender	Risk group	WBC count at diagnosis (× 10^9^/L)	Immunophenotype	Karyotype	*BCR::ABL1*-like rearrangement; Fusion partner (percentage of cells)	*JAK2* exon 16 status	Response to induction	Clinical outcome	Status dead/alive
31/F	SR	9,44	B-common	47,XX + mar[4]/46,XX[12]	*CRLF2*-r Fusion partner not identified (70% of cells)	Non-mutated	CR MRD negative	Relapse after maintenance therapy	Dead
21/M	SR	21,22	B-common (expression of CRLF2: 79%)	ND	*CRLF2*-r *CRLF2::IGH* (80% of cells)	Non-mutated	CR MRD negative	Sudden death in CR1 during consolidation—pulmonary embolism	Dead
55/M	HR	200	B-common (expression of CRLF2: 100%)	ND	*CRLF2*-r *CRLF2::IGH* (50% of cells)	Non-mutated	CR MRD negative	Death after induction—MODS, PRES	Dead
32/M (19 at initial diagnosis)	HR	14,5	B-common (expression of CRLF2: 99%)	45,XY,del(9)(p21),der(19)(:19p13.2 → 19q13.4::?),-20[9]/45,idem,der(17)(?::17p11.2 → 17qter)[2]/45,idem,der(3)(3pter → 3q13.3::?),-14, + der(19)(:19p13.2 → 19q13.4::?)[1]/45,idem,der(3)(3pter → 3q13.3::?)[1],44,idem,-14,der(17)( ?::17p11.2 → 17qter)[1]/44,idem,-14[1]/46,XY,der(3)(3pter → 3q13.3::?),del(9)(p21),add(14)(qter)[1]/46,XY,der(3)(3pter → 3q13.3::?)[1].nuc ish(CRLFx2)(5’CRLF sep 3’CRLFx1)[40/50],(IGHx2)(3’IGH sep 5’IGHx1)[98/100],(PBX1 × 2,HLFx2,E2Ax1)[43/50]	*CRLF2*-r *CRLF2::IGH* (75% of cells)	*JAK2* c.2049A>C (p.R683S)	CR MRD negative	alloHSCT in CR1; relapse after 13 years; *CRLF2*-r diagnosed during relapse; CR2 MRD negative after reinduction	Alive
32/M	HR	127	Pro-B	ND	*ABL1*-r Fusion partner not identified (15% of cells)	NA	CR MRD positive	alloHSCT in CR1; relapse after 3 months post-alloHSCT	Dead
29/M	HR	55,1	B-common	41–45,XY,-6[2],-18[3],-20[3],-21[2],-22[3][cp10]/46,XY[11]	*ABL2*-r Fusion partner not identified (30% of cells)	NA	CR MRD positive	alloHSCT in CR1	Alive

*No* number, *F*female, *M*male, *BCR::ABL-like ALL* BCR::ABL-like acute lymphoblastic leukemia, *CRLF2-r* CRLF2-rearranged, *ABL1-r* ABL1-rearranged, *ABL2-r* ABL2-rearranged, *ND* no data, *NA* not analyzed, *CR* complete remission, *MRD* minimal residual disease, *MODS* multiple organ dysfunction syndrome, *PRES* posterior reversible encephalopathy syndrome, *alloHSCT* allogeneic hematopoietic stem cell transplantation

**Fig. 4:**
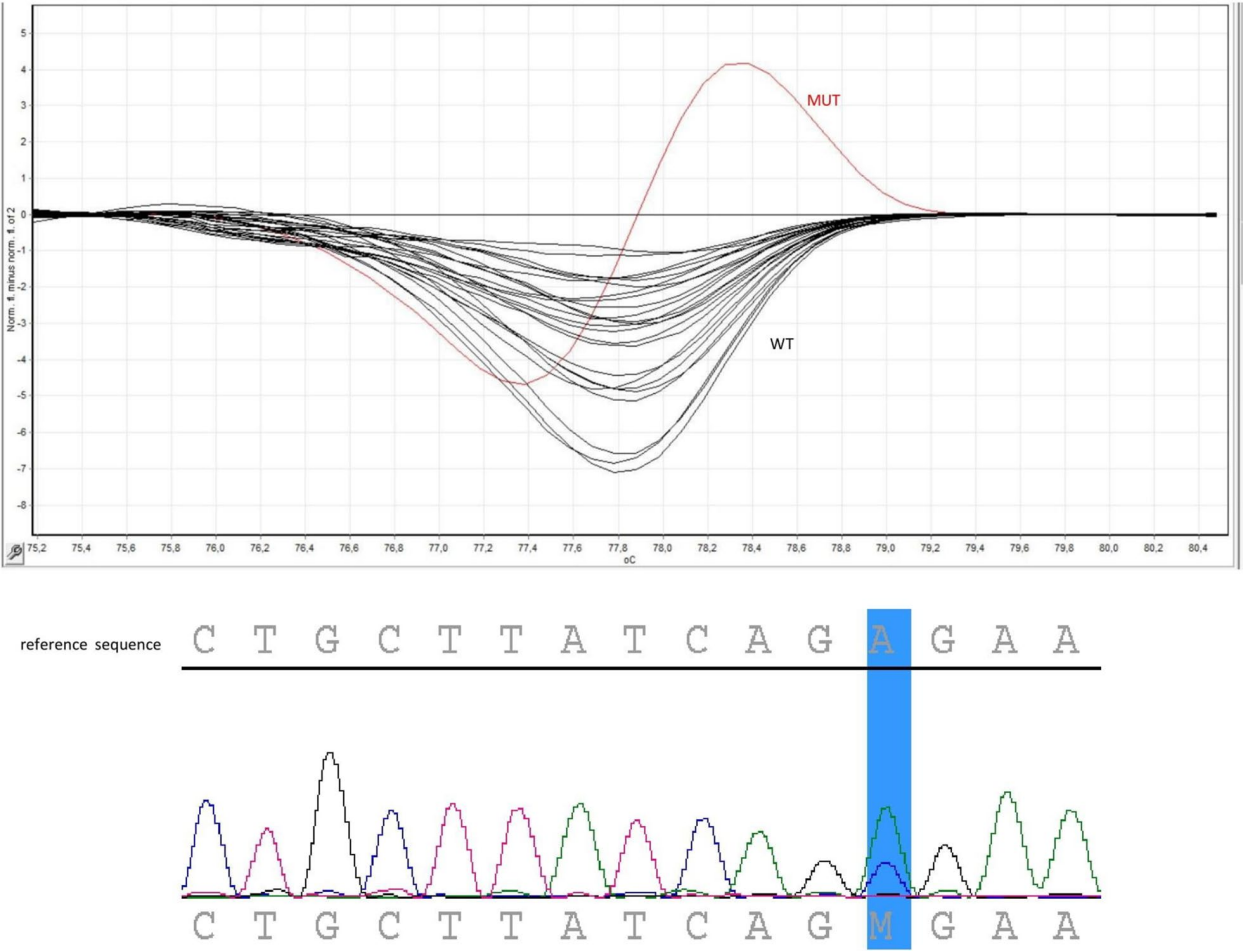
The high resolution melt analysis (HRMA)—top field, and Sanger sequencing result (bottom field) in the *CRLF2*-r patients. HRMA revealed abnormal melting profile in one studied sample, in contrast to the normal double-stranded DNA dissociation characteristics during heating in control samples (wild type, WT). In the presented case, Sanger sequencing study revealed the presence of the variant LRG_612:c.2049A>C(p.Arg683Ser). Reference transcript ID (RefSeq): NM_004972.4:c.2049A>C, NP_004963.1:p.(Arg683Ser)

Additionally, patients with *CRLF2*-r were enrolled in the analysis of the *JAK2* exon 16 mutational status. HRMA revealed different melting profile in one studied sample (*CRLF2*-r case). We confirmed the presence of the variant LRG_612:c.2049A>C(p.Arg683Ser) using Sanger sequencing in this case (Fig. 4). Overall, the incidence of point mutation in the *JAK2* exon 16 within *CRLF2*-r cases was 25%.

## Discussion

Herein we present a strategy to identify cases with potentially targetable genomic lesions which can be applied in a limited resource setting. A similar approach integrating FISH and FCM has been implemented by Sharma and Virk [20, 21]. In the material from our Department, high expression of CRLF2 was indicative of the presence of *CRLF2*-r, similarly to observations from a larger cohort in the study of Virk et al. [21]. The frequency of *BCR::ABL1*-like cases in our material was 15.1% of B-ALL patients. The incidence of this entity in our cohort appears to be lower than in the literature data [6, 7]. It might be the result of both substantial number of cases excluded lacking adequate material and of limited techniques applied in the study. Sharma used a similar cost-effective approach in a larger cohort and revealed a slightly lower incidence of *BCR::ABL1*-like cases in − 11.4% of B-ALL in the screened group. Notably, this study group included adults, as well as the pediatric population, in which *BCR::ABL1-like* ALL is less frequently reported [20]. Among JAK-STAT pathway fusions, the rearrangements of *CRLF2* account for the majority of cases [8, 9, 20]. The overexpression of *CRLF2* observed in FCM, may be the result of either cryptic deletion of the pseudoautosomal region 1 of chromosomes X and Y leading to the gene fusion *P2RY8::CRLF2*, or the translocation resulting in the gene fusion *IGH::CRLF2* [21, 22]. Approximately 50% of patients with *CRLF2*-r ALL harbor mutations in the *JAK* family genes, mainly in the *JAK2* gene [6]. In our group, one patient harbored a point mutation within the exon 16 of the *JAK2* gene, *JAK2* c.2049A>C (p.R683S), accounting for 25% of *CRLF2*-r cases. Notably, the mutation occurred in the patient with a relapse after a prolonged remission post-alloHSCT. The relatively low frequency of *JAK2* mutations in the study may be explained by the applied technique, which is less sensitive than the next generation sequencing implemented in numerous reports, a small study group and the fact that *JAK2* mutations in the *BCR::ABL1*-like ALL may occur in other coding regions.

The optimal treatment strategy of the *BCR::ABL1*-like ALL is debatable. As far as the molecular background of this subtype is concerned, the combination of standard chemotherapy with TKI remains promising. Several preclinical studies and case studies reported safety, activity and efficacy of the JAK inhibitor, ruxolitinib, in *BCR::ABL1*-like ALL harboring JAK-STAT-activating aberrations and ABL-class inhibitors in cases with rearrangements of ABL-class genes [6, 10, 14, 23–28]. The studies by Steeghs et al., on the other hand, revealed that proliferation of *JAK2* mutated ALL cells depended on several signaling pathways activity [29]. Hence, while *JAK2*-r leukemic cells were found to be susceptible to *JAK* inhibitors, both ruxolitinib and momelotinib, the efficacy of JAK specific therapy may be limited in *JAK2* mutated cells. Similar results were observed by Schwartzman et al. [30]. Furthermore, the study of Steeghs et al. provides rationale for the hypothesis that *JAK2* mutations may be secondary lesions in the leukemic process, while *JAK2* rearrangements are leukemic drivers. Therefore, it is suggested to combine JAK inhibitors with Ras pathway inhibitors to avoid clonal selection [29–32]. A synergistic effect of combination of TKIs with antagonists of the BCL-2 anti-apoptotic protein, venetoclax and navitoclax, was also reported [24].

The role of alloHSCT in the first CR is also a subject of debate, since the prognostic impact of MRD negativity post-induction remains questioned [33, 34]. An analysis of Koller et al. suggests that alloHSCT may overcome the poor prognosis of *CRLF2*-r ALL [35]. It is postulated that patients with the presence of *CRLF2*-r and *JAK2*-r should be considered as candidates for alloHSCT [36]. On the other hand, relapses post-alloHSCT are often driven by *CRLF2*-r clones. These relapses occur irrespective of the MRD-negativity achievement, since *CRLF2* fusions are considered early events in the leukemogenesis and *CRLF2*-r malignant clone may persist in a quiescence during the treatment, and eventually escape the immune system or gain a proliferative state trough acquired mutations [37–39]. Notably, herein we report a case of a patient with *CRLF2*-r which occurred during a relapse after a prolonged remission, despite the absence of *CRLF2*-r in at the initial diagnosis. Conversely, we could not exclude the possibility of overexpression of CRLF2 on leukemic blast at the original diagnosis, since it was not evaluated in flow cytometry at that time. Shah et al. described a similar case of an individual with a relapse of *CRLF2*-r ALL after a prolonged remission, however, the authors did not analyze the presence of *CRLF2*-r in the material from the initial diagnosis [39]. Studies by Aldoss et al. revealed that during a late relapse of ALL after alloHSCT, novel cytogenetic aberrations might occur as a manifestation of a genetic evolution of the disease or clonal selection, or even due to de novo secondary leukemogenesis related to former therapy [40].

Although our study provided valuable results regarding *BCR::ABL1*-like ALL diagnosis, it did have some drawbacks. The first is the small number of patients enrolled in the study and its mainly retrospective nature due to a relatively low incidence of B-ALL in the adult population. A substantial proportion of patients was eventually excluded as a result of lack of adequate cytogenetic material, which could impact the overall incidence of *BCR::ABL1*-like cases in the analyzed group. The second limitation is the use of standard diagnostic techniques which, however, are acceptable, if next generation techniques are unattainable [41]. Another limitation of the study is the fact that enrolled patients were treated over a long time period of time. Although most of the subjects were diagnosed and treated according to the guidelines of PALG, the therapeutic protocol evolved over the last decade, hence the patients were not uniformly treated. Finally, this is a single-center study, therefore, it presents a cohort which is not large enough to show significance. On the other hand, our results remain useful for future meta-analysis on *BCR::ABL1*-like ALL incidence and outcomes from real-world settings. Our study demonstrates that smaller centers can potentially provide useful information regarding *BCR::ABL1*-like ALL, regardless of the limited techniques employed. Our results remains also essential considering the potential advent of molecularly targeted therapy in *BCR::ABL1*-like patients.

## Conclusions

The diagnostic strategy implementing widely available techniques enables the identification of high risk and therapeutically targetable cases of *BCR::ABL1*-like ALL. The presented approach may be particularly appropriable in settings with limited resources.

## Data Availability

Data available upon request from the authors.
